# Comprehensive Analysis of the Immune and Stromal Compartments of the CNS in EAE Mice Reveal Pathways by Which Chloroquine Suppresses Neuroinflammation

**DOI:** 10.3390/brainsci10060348

**Published:** 2020-06-05

**Authors:** Rodolfo Thome, Alexandra Boehm, Larissa Lumi Watanabe Ishikawa, Giacomo Casella, Jaqueline Munhoz, Bogoljub Ciric, Guang-Xian Zhang, Abdolmohamad Rostami

**Affiliations:** Department of Neurology, Thomas Jefferson University, Philadelphia, PA 19107, USA; rodolfo.thome@jefferson.edu (R.T.); alexandra.boehm@jefferson.edu (A.B.); larissa.ishikawa@jefferson.edu (L.L.W.I.); giacomo.casella@jefferson.edu (G.C.); jlima.munhoz@gmail.com (J.M.); bogoljub.ciric@jefferson.edu (B.C.); guang-xian.zhang@jefferson.edu (G.-X.Z.)

**Keywords:** chloroquine, EAE, dendritic cells, microglia, astrocytes, oligodendrocytes

## Abstract

Multiple sclerosis (MS) and experimental autoimmune encephalomyelitis (EAE) are neuroinflammatory diseases of the central nervous system (CNS), where leukocytes and CNS resident cells play important roles in disease development and pathogenesis. The antimalarial drug chloroquine (CQ) has been shown to suppress EAE by modulating dendritic cells (DCs) and Th17 cells. However, the mechanism of action by which CQ modulates EAE is far from being elucidated. Here, we comprehensively analyzed the CNS of CQ and PBS-treated EAE mice to identify and characterize the cells that are affected by CQ. Our results show that leukocytes are largely modulated by CQ and have a reduction in the expression of inflammatory markers. Intriguingly, CQ vastly modulated the CNS resident cells astrocytes, oligodendrocytes (OLs) and microglia (MG), with the latter producing IL-10 and IL-12p70. Overall, our results show a panoramic view of the cellular components that are affect by CQ and provide further evidence that drug repurposing of CQ will be beneficial to MS patients.

## 1. Introduction

MS and EAE are inflammatory diseases of the CNS. Although the mechanisms that trigger MS are not fully elucidated, studies in EAE have uncovered the major role played by T cells in disease development, severity, and recovery [1]. Inflammatory CD4^+^ T cells differentiate into IFN-γ^+^ Th1 cells and IL-17^+^ Th17 cells and migrate to the CNS where they induce inflammation and the chemoattraction of myeloid cells [2,3,4,5]. We and others have shown that the cytokine GM-CSF is an essential mediator of pathogenic Th17 cells [6,7,8]. Moreover, GM-CSF is induced in Th17 cells by IL-23R stimulation [7,9]. Conversely, Foxp3-expressing regulatory T (Treg) cells suppress Th1/Th17 cells and overall inflammation through contact-dependent and independent mechanisms [10]. Additionally, Treg cells were shown to promote remyelination [11,12]. In this context, antigen-presenting cells, such as dendritic cells (DCs), may direct T cell differentiation to Th17/Th1 and Treg cell phenotypes [13,14,15].

CNS resident cells are affected by local inflammation and may contribute to neurodegeneration in EAE. At early stages of EAE, microglia (MG) acquire an inflammatory M1 profile with the production of IL-1β, IL-6 and nitric oxide (NO), which shifts towards a tissue repair-associated M2 phenotype at later stages [16,17,18]. MG-derived IL-1β promote neuroinflammation and MG proliferation in an autocrine manner [19]. Moreover, MG-astrocyte crosstalk greatly influences the outcome of neuroinflammation in EAE [20,21]. Inflammatory A1 astrocytes actively induce damage to oligodendrocytes (OLs) and neurons, promote leukocyte infiltration and aid neuroinflammatory processes [22,23]. These observations illustrate the complex network and interactions among peripherally-derived leukocytes and CNS resident cells in EAE and MS. Thus, drugs that target multiple cell types while inducing tissue repair are of great importance in MS therapy.

Chloroquine (CQ) is a known antimalarial drug with anti-inflammatory properties [24]. CQ modulates monocyte activation and TNF-α production [25,26]. CQ also inhibits lysosomal maturation and antigen-processing [27,28,29]. By modulating DCs and Th17 cells, CQ has been shown to suppress EAE [27,30,31]. However, the mechanisms by which CQ suppresses EAE are far from being elucidated. CQ induces tolerogenic DCs in a STAT1-dependent manner [32]. Interestingly, CQ inhibited Th17 cell differentiation in a STAT-1-independent and T-bet-dependent fashion [31]. These observations suggest that CQ interferes with multiple cell types and influences diverse signaling pathways to suppress inflammation. The identification of such cell types and pathways may provide a better understanding of the mechanisms by which CQ suppresses EAE and may shed light on new therapeutic targets in MS.

In this study, we comprehensively characterized the phenotype and activity of peripherally derived leukocytes and CNS resident cells in CQ-treated EAE mice. We observed that CQ suppressed inflammatory leukocytes while also modulating MG and astrocytes. Further analyses revealed that CQ reduced IL-23R expression in CD4^+^ T cells and IL-23 production by MG. Moreover, CQ stimulated MG to produce IL-10 and IL-12p70, which stimulated IL-10 production in T cells and refrained astrocyte activation. Together, our results show that CQ has a broad action by modulating leukocytes and CNS resident cells to suppress EAE and provide further evidence that drug repurposing of CQ is beneficial to patients with MS.

## 2. Methods

### 2.1. Animals

We used 8–12 week old male and female C57BL/6 and B6SJLF1 mice from the Jackson Laboratory (Bar Harbor, ME, USA). Mice were acclimated in clean cages in a controlled environment with food and water ad libitum. Experiments detailed in this study were approved by Thomas Jefferson University’s IACUC under protocol numbers 01970 and 02034.

### 2.2. EAE Induction and Evaluation

To induce and evaluate EAE, we followed previously described protocols [33,34]. Mice were subcutaneously immunized with 200 µg of MOG_35-55_ peptide (Genscript) and equal volume of Complete Freund’s adjuvant supplemented with 10 mg/mL of heat-killed *Mycobacterium tuberculosis* H37Ra. Additionally, mice were intraperitoneally injected with 240 ng of Pertussis toxin at 0 and 2 days after immunization. EAE development was analyzed daily and scored on a 0–5 scale, where: 0—no clinical sign, 1—limp tail, 2—hind paw weakness, 3—hind paw paralysis, 4—hind paw paralysis and front paw weakness, 5—full paralysis/death.

### 2.3. CQ Treatment

The dosage for CQ treatment has been assessed before [35]. Mice were treated with CQ (chloroquine diphosphate salt, Sigma-Aldrich) at a 5 mg/kg concentration via i.p. injections. The pH in CQ solution was 7.2. Control mice were injected with diluent solution (phosphate-buffered saline 0.02 M pH 7.2).

### 2.4. Isolation of Mononuclear Cells in the CNS of Mice with EAE

Mononuclear cells from the CNS of EAE mice were isolated by Percol gradient centrifugation following published reports [32,33,34]. In brief, euthanized mice were perfused with ice-cold PBS and the CNS tissue was collected and incubated with 700 µg/mL Liberase TL (Sigma-Aldrich, St. Louis, MO, USA) at 37 °C for 30 min. To remove myelin debris, the digested tissue was centrifuged in a 30% Percol solution. MNCs were recovered from the bottom of the tube and used for flow cytometry analyses.

### 2.5. Flow Cytometry

For detection of intracellular cytokines by flow cytometry, cells were stimulated with PMA (50 ng/mL), ionomycin (500 ng/mL) and GolgiPlug (1 µg/mL) in IMDM complete medium for 3 h at 37 °C. Cells were washed in FACS buffer (PBS/2% FBS) and stained with fluorochrome labeled Abs to surface molecules for 20 min at 4 °C. Cells were then fixed and permeabilized (Invitrogen/ThermoFisher, Waltham, MA, USA) and incubated with antibodies against intracellular antigens for 18 h at 4 °C. Immediately before acquisition, cells were washed and resuspended in PBS. We utilized a FACSAria Fusion (BD Biosciences) flow cytometer for acquisition and FlowJo VX (Tristar Inc., Ashland, OR, USA) for analyses. Antibodies used in this study were anti-mouse: CD45 (30-F11), TCR-β (H57-597), CD4 (GK1.5), CD8 (53-6.7), GFAP (2E1.E9), CD11b (M1/70), Ly6C (HK1.4), CD11c (N418), MHC-II (M5/114.15.2), CD80 (16-10A1), CD86 (GL-1), pSTAT1 (A15158B), pSTAT3 (13A3-1), mTOR (O21-404, from BD Biosciences), IL-1β (NJTEN3, from eBioscience/ThermoFisher), IL-6 (MP5-20F3), IL-10 (JES5-16E3), IL-12p70 (C15.6, from BD Biosciences), IL-17A (TC11-18H10.1), IL-23 (N71-1183, from BD Biosciences), GM-CSF (MP1-22E9), Foxp3 (FJK-16s, from eBioscience/ThermoFisher, Waltham, MA, USA), IL-23R (12B2B64), IL-10R (1B1.3a), Granzyme B (GB11), and IRF8 (V3GYWCH, from eBioscience/ThermoFisher). All antibodies used in this study were purchased from Biolegend, San Diego, CA, USA, except where mentioned otherwise. 

### 2.6. Isolation of Primary MG and CQ Treatment

CD11b^+^ MG were isolated from MNCs obtained from the CNS of P0–P3 pups using magnetic beads (Miltenyi Biotec., Auburn, CA, USA). This isolation procedure yielded a consistent purity of 95% of CD11b^+^ cells assessed by flow cytometry. MG were activated with LPS (100 ng/mL) with or without CQ (50 µM) for 18 h at 37 °C. The optimal CQ concentration for in vitro treatment of myeloid cells has been determined before [27]. At the end of culture time, MG cells were processed for flow cytometry, RNA extraction and co-culture.

### 2.7. PCRArray and Gene Ontology Analysis

RNA was extracted and reverse-transcribed from primary MG utilizing commercially available kits (RNAeasy extraction kit and high capacity RNA-to-cDNA kit, respectively, both from ThermoFisher). The cDNA was tested for quality and purity in a nanodrop equipment before being subjected to PCRArray (ThermoFisher). Gene ontology analysis was performed with CytoScape v3.8 (CytoScape.org).

### 2.8. Co-Culture of MG and T Cells

Primary MG were treated as above and extensively washed with Iscove’s Modified Dulbecco Medium (IMDM) to remove LPS and CQ from the cells. In total, 50,000 MG were seeded into each well of a 96 U-bottom well plate. Then, CD4^+^ T cells were isolated from spleens of naïve mice using magnetic beads (Miltenyi Biotec) and 100,000 cells were seeded on each well on top of MG. As controls, T cells were cultured without MG. As stimulus, cells were incubated with agonistic anti-CD3 antibody at a concentration of 0.5 µg/mL. For these analyses, co-stimulation was provided by MG without the need for anti-CD28 antibodies. Cells were incubated for 72 h at 37 °C as described [32], and then, analyzed by flow cytometry.

### 2.9. Co-Culture of MG and Astrocytes

Primary MG were treated as above and extensively washed with Iscove’s Modified Dulbecco Medium (IMDM) to remove LPS and CQ from the cells. In total, 50,000 MG were seeded into each well of a 96 U-bottom well plate. Then, 50,000 C8D30 astrocytes (ATCC-CRL-2534) were seeded on each well on top of MG. As controls, astrocytes were cultured without MG. Cells were incubated for 48 h at 37 °C before being collected and analyzed by flow cytometry.

### 2.10. Statistical Analyses

Daily clinical scores among experimental groups in EAE were compared by two-way ANOVA and post-tested with Sidak. Comparisons between two groups were carried out with unpaired Student’s *t* test and Welch’s correction. Values of *p* < 0.05 were defined as significant.

## 3. Results

### 3.1. CQ Reduces Ongoing Inflammation in Relapsing EAE

**Figure 1:**
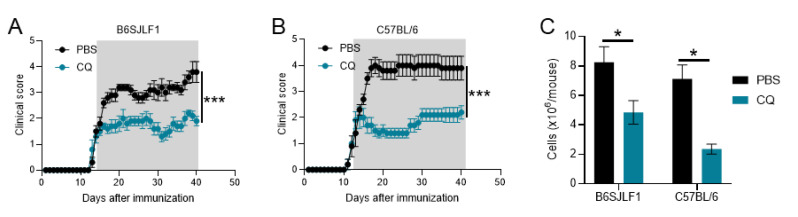
Chloroquie (CQ) suppresses neuroinflammation in two models of experimental autoimmune encephalomyelitis (EAE). C57BL/6 and B6SJLF1 mice (*n* = 5/group) were immunized to induce EAE. At the onset and until the end of experimentation, mice were treated with CQ (5 mg/kg) via i.p. everyday. (**A**) Disease development in B6SJLF1 mice and in (**B**) C57BL/6 mice. (**C**) CNS cells were analyzed at day 21 post-immunization. Values of *p* < 0.05 (*) and < 0.001 (***) were considered statistically significative. Results are from three independent experiments.

CQ has been shown to prevent EAE development when given prophylactic and to reduced EAE severity when given at disease onset [31,35]. However, if CQ is able to suppress ongoing relapsing EAE is unknown. B6SJLF1 mice, the offspring of C57BL/6 and SJL mice, develop relapsing EAE when immunized with MOG_35-55_ [36,37]. We immunized mice to develop EAE and CQ treatment started at the onset of clinical signs of EAE. Mice treated with PBS fully developed disease clinical signs and the characteristic relapsing feature of this model (Figure 1A). However, mice treated with CQ developed a significantly less severe EAE compared with PBS-treated mice (Figure 1A). These results are on par with those observed in chronic EAE in C57BL/6 mice (Figure 1B). B6SJLF1 mice treated with CQ had a significant decrease in the infiltration of leukocytes to the CNS compared with those treated with PBS (Figure 1C). A similar trend was observed in treated C57BL/6 mice (Figure 1C). These results show that CQ is a potent agent in suppressing the clinical development of two models of EAE.

### 3.2. Peripherally-Derived Leukocytes Are Modulated in CQ-Treated Mice

**Figure 2:**
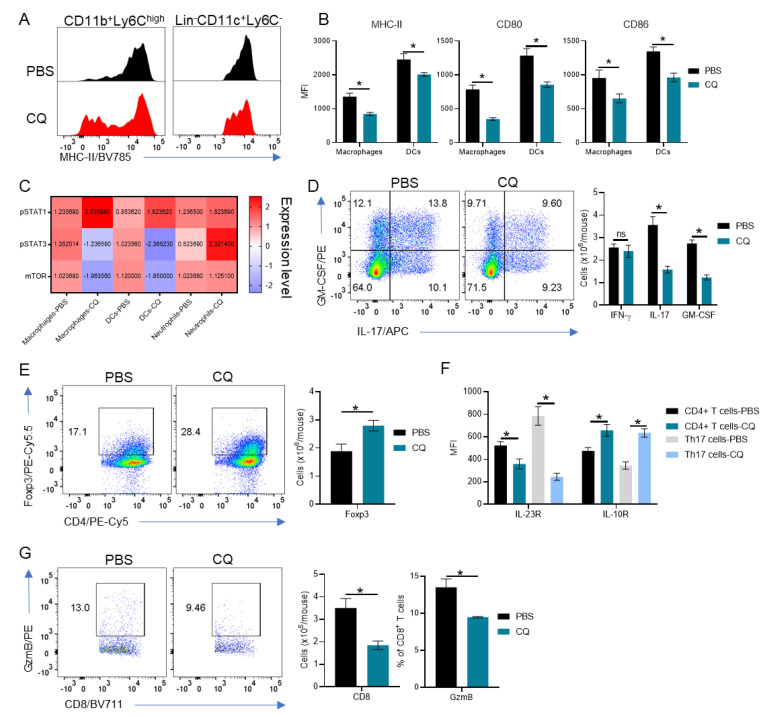
Comprehensive analysis of the immune compartment in the central nervous system (CNS) of CQ-treated mice. CNS cells from EAE B6SJLF1 mice (*n* = 5/group) at day 21 post-immunization were analyzed by flow cytometry. (**A**) Representative histogram for MHC-II expression in CD11b^+^Ly6C^high^ macrophages and in Lin^-^CD11c^+^Ly6C^-^ DCs. (**B**) MHC-II, CD80 and CD86 expression in macrophages and dendritic cells (DCs). (**C**) Mean fluorescent values for pSTAT1, pSTAT3 and mTOR in macrophages, DCs and Ly6G^+^ neutrophils were measured by flow cytometry. (**D**) Infiltrating GM-CSF-, IFN-γ and IL-17-producing CD4^+^ T cells in the CNS of EAE mice. (**E**) Numbers of Foxp3^+^ Treg cells. (**F**) Analysis of IL-23R and IL-10R in total CD4^+^ T cells and in Th17 cells in the CNS of EAE mice. (**G**) Analysis of total CD8^+^ T cell GzmB-producing CD8^+^ T cell infiltration in the CNS of EAE mice. Values of *p* < 0.05 (*) were considered statistically significative. Ns: not significative. Results are from three independent experiments.

We analyzed the phenotype of leukocytes in the CNS of CQ-treated EAE B6SJLF1 mice. We observed a significant reduction in numbers of CD45^high^ leukocyte in mice treated with CQ compared with those treated with PBS (Figure 1C). Furthermore, DCs and macrophages from CQ-treated mice presented a reduced expression of the molecules involved in antigen-presentation and activation: class II MHC, CD80, CD86 in comparison with controls (Figure 2A). Analyses of the signaling mediators pSTAT1, pSTAT3, and mTOR in peripherally derived myeloid cells from CQ-treated mice revealed a decrease in the expression of pSTAT3 and mTOR in DCs and macrophages, but not in neutrophils in comparison with those from PBS-treated mice (Figure 2C). pSTAT1 was significantly upregulated in DCs and macrophages while pSTAT3 was upregulated in neutrophils (Figure 2C). There was no significant difference in mTOR expression in neutrophils between the two groups of mice (Figure 2C).

We also observed a significant decrease in IL-17^+^ and GM-CSF^+^CD4^+^ T cells in the CNS of CQ-treated mice compared with PBS-treated ones (Figure 2D). No significant differences were observed in Th1 cells (Figure 2D). Foxp3^+^ Treg cells were upregulated in CQ-treated mice compared with controls (Figure 2E). Interestingly, we observed a significant decrease in the expression of IL-23R and an increase in the expression of IL-10R in total CD4^+^ T cells and more drastically in Th17 cells (Figure 2F). CD8^+^ T cells numbers were reduced and had lower expression of Granzyme B in CQ-treated mice compared with PBS-treated mice (Figure 2G). B cells did not show significant differences in numbers, MHC-II expression and in cytokine production (not shown). Combined, these results show that CQ modulates both myeloid and lymphoid leukocyte populations in the CNS of EAE mice.

### 3.3. MG and Astrocytes Are Modulated by CQ

**Figure 3:**
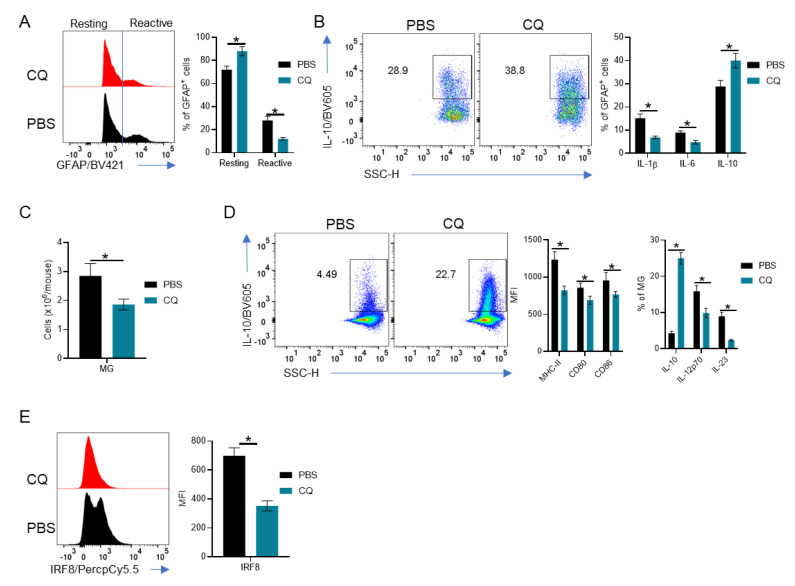
Comprehensive analysis of the stromal compartment in the CNS of CQ-treated mice. CNS cells from EAE B6SJLF1 mice (*n* = 5/group) at day 21 post-immunization were analyzed by flow cytometry. (**A**) Analysis of resting (GFAP^+/low^) and reactive (GFAP^high^) astrocytes. (**B**) Analysis of IL-1β, IL-6 and IL-10 production by astrocytes. (**C**) Absolute numbers of MG (CD45^+/low^CD11b^+^Ly6C^-^) in the CNS of CQ- and PBS-treated mice. (**D**) Analysis of the expression of the antigen-presenting molecules MHC-II, CD80 and CD86 and of the cytokine profile of MG. (**E**) IRF8 expression in MG from CQ- and PBS-treated mice. Values of *p* < 0.05 (*) were considered statistically significative. Results are from three independent experiments.

We then analyzed the effect of CQ treatment on CNS resident cells of EAE mice. We observed that GFAP^+^ astrocytes presented as two populations GFAP^+^ and GFAP^high^ (Figure 3A), which reflect their activation profile. GFAP^high^ are reactive astrocytes that are involved in scar formation and oligodendrocyte death [38,39]. We observed a significant decrease in GFAP^high^ astrocytes in CQ-treated mice compared with PBS-treated controls (Figure 3A). Moreover, astrocytes from CQ-treated mice showed an increase in IL-10 production and a decrease in IL-6 and IL-1β production in comparison with cells from PBS-treated mice (Figure 3B).

Furthermore, MG greatly influences astrocyte activation [40,41] and provides additional antigen stimulation to T cells in the CNS [42,43,44]. Thus, we investigated the phenotype of MG in mice treated with CQ. Our results showed that the numbers of MG were reduced in the CNS of CQ-treated mice compared with those from PBS-treated mice (Figure 3C). Interestingly, MG from CQ-treated mice had an increase in IL-10 and IL-12p70 cytokine production while MHC-II, CD80 and CD86 levels were decreased in comparison with MG from PBS-treated mice (Figure 3D). Moreover, we observed a significant decrease in IL-23 production in MG from CQ-treated mice compared with controls (Figure 3D). The transcriptional factor IRF8, which promotes MG differentiation and aids in IL-1β production by MG [45,46,47,48], was significantly reduced in MG from CQ-treated mice (Figure 3E). Together, these results reveal a portrait of CNS resident cells in EAE mice treated with CQ, where astrocytes and MG acquire an immunomodulatory phenotype.

### 3.4. CQ Induces IL-10 and IL-12p70 in MG Which Enhances IL-10 Production by T Cells and Reduces Astrocyte Activation

**Figure 4:**
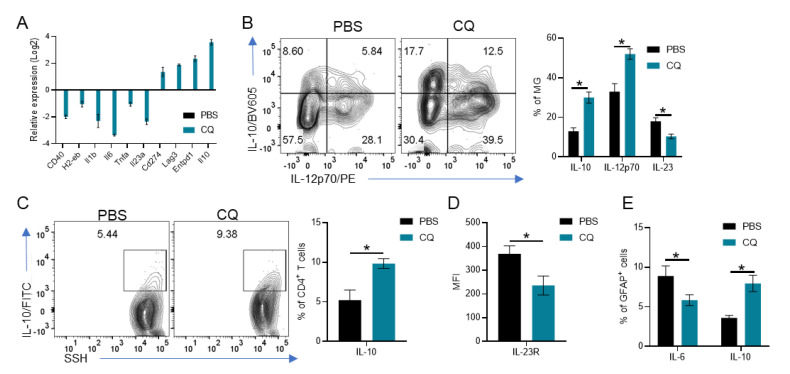
CQ-treated MG modulates T cells and astrocytes. Primary MG were activated with LPS (100 ng/mL) in the presence or absence of CQ (50 µM) for 18 h. (**A**) Gene expression analysis in CQ-treated MG compared with PBS-treated ones. (**B**) Analysis of IL-10, IL-12p70 and IL-23 production by MG. (**C**) MG were co-cultured with CD4^+^ T cells and the production of IL-10 was analyzed by flow cytometry. (**D**) IL-23R in CD4^+^ T cells cultured with MG. (**E)** MG were cultured with C8D30 astrocytic cells and the production of IL-6 and IL-10 was analyzed by flow cytometry. Cultures were carried out in triplicate. Values of *p* < 0.05 (*) were considered statistically significative. Results from three independent experiments.

Finally, we investigated whether CQ modulates MG directly. We isolated primary MG and activated the cells with LPS for 18 h in the presence or absence of CQ. Then, the RNA from MG was analyzed by PCRArray. We observed that CQ reduced the expression of genes associated with inflammation (Cd40, H2-eb, Il1b, Il6, Tnfa, Il23a), while increasing those related to immune modulation (Il10, Lag3, Entpd1, Cd274) (Figure 4A). Moreover, CQ-treated MG produced significantly more IL-10 and IL-12p70 and less IL-23 than PBS-treated MG (Figure 4B).

To test their effect on CD4^+^ T cell activation, we co-cultured CQ-treated MG with CD4^+^ T cells isolated from naïve WT mice. We observed that CQ-treated MG induced significantly more IL-10 production in T cells than PBS-treated MG (Figure 4C). Moreover, IL-23R in T cells cultured with CQ-treated MG was downregulated when compared with T cells cultured with PBS-treated MG (Figure 4D).

To test the effect of CQ-treated MG on astrocyte activation, we co-cultured MG with the astrocytic cell line C8D30. We observed that CQ-treated MG induced a significant increase in IL-10 production by astrocytes, while PBS-treated MG induced higher IL-6 production (Figure 4E). Collectively, our results show a pivotal role of CNS resident cells, especially MG, on CQ-induced immunosuppression in EAE.

## 4. Discussion

In this study, we show that CQ modulates a broad array of cell subtypes to reduce EAE severity. Although the therapeutic effect of CQ in EAE was shown before, its underlying mechanism of action remains to be fully elucidated. Here, our results have uncovered a portrait of the CNS resident and infiltrating cells after CQ treatment. We observed that CQ reduced the numbers and phenotype of inflammatory leukocytes and also upregulated the expression of modulatory mediators in leukocytes and CNS resident cells.

EAE is a T cell-dependent model of MS, where pathogenic Th1 and Th17 cells play a major role in disease severity and development [1]. In addition, GM-CSF production grants pathogenicity of Th17 cells [7]. Thus, we investigated the profile of CD4^+^ T cells in CQ-treated mice and compared them with those found in PBS-treated mice. Our results showed that CQ inhibited differentiation of Th17 and GM-CSF^+^ CD4^+^ T cells without disturbing the frequencies of Th1 cells. This finding is in line with a recent publication from our group, where we show that Th17 cells are more sensitive to CQ treatment [31]. Moreover, CQ reduces the differentiation of Th17 cells by inducing the expression of T-bet [31]. GM-CSF production in Th17 cells is induced through stimulation of the IL-23 receptor [7,9]. Interestingly, CQ reduced the expression of IL-23R on CD4^+^ T cells. These results show that CQ modulates T cells through different mechanisms: either by suppressing their differentiation into inflammatory Th17 cells or by reducing their pathogenicity through downregulation of IL-23R.

We also observed a reduction in CD8^+^ T cells numbers in the CNS of CQ-treated mice. Although EAE is mainly a Th cell model, CD8^+^ T cells were shown to play an important role shaping the phenotype of CD4^+^ T cells during EAE through the Qa-1 receptor [49,50]. In fact, pathogenic MOG-reactive CD8^+^ T cells cooperate with Th cells to promote sustained CNS inflammation in EAE [51], whereas dual modulation of CD8^+^ and CD4^+^ T cells proved to be more efficient in suppressing EAE [52]. Our results showed that CQ induced suppression of both CD8^+^ and CD4^+^ T cells, which may account for its efficiency in reducing EAE severity.

We have showed previously that CQ induces Foxp3^+^ Treg cells indirectly by stimulating tolerogenic DCs [35]. Additionally, CQ directly modulates DCs by inducing iNOS and pSTAT1 expression, and the lack of these molecules abrogated the tolerogenic phenotype of CQ-treated DCs [30,32]. A similar increase in Foxp3^+^ Treg cells in models of inflammatory bowel disease and lupus was observed [53,54]. CQ directly induced Foxp3 expression in T cells in a Nurr1-dependent manner [53]. Thus, the literature data show that CQ induces STAT1 in DCs, T-bet in Th17 cells and Nurr1 in naïve T cells [32,53,54]. In line with this observation, CQ modulates tumor infiltrating macrophages through stimulation of the NF-kB signaling pathway [29]. In the present study, we show that STAT3 and mTOR signaling pathways in myeloid cells were also affected by CQ. Specifically, pSTAT1 was upregulated in DCs and macrophages, while pSTAT3 was upregulated in neutrophils. mTOR was decreased in DCs and macrophages, and unaffected in neutrophils. These results show that CQ induces different signaling cascades in various leukocytes.

MG are CNS resident cells that closely resemble peripheral macrophages [55,56]. MG act as a first line defense in the CNS against infections [16,21,46]. In EAE, MG cooperate with local inflammation and provide antigen-stimulation to infiltrating T cells [40,43]. Thus, we analyzed MG phenotype in CQ-treated mice, and we found that CQ inhibited MG activation. Moreover, we observed a reduced production of IL-23 and an increase in IL-10 and IL-12p70 cytokines in MG from CQ-treated mice. CQ modulated MG directly in in vitro experiments and conferred an anti-inflammatory profile in them. In co-culture experiments with total CD4^+^ T cells, CQ-treated MG reduced GM-CSF production and increased IL-10 production. These results are in line with our in vivo observations, where CD4^+^ T cells had a reduced GM-CSF and increased IL-10 production while IL-23R expression was also reduced.

In prophylactical treatment with CQ, it is possible that EAE amelioration relied on the modulation of antigen-presenting cells, such as DCs and macrophages [27,35]. However, during ongoing EAE, there is a possibility that CQ acts directly on Th17 cells in the CNS and on MG to refrain further T cell activation and promote an immunosuppressive microenvironment [31,57,58]. In line with this, MG modulated T cells and astrocytes. Astrocytes cultured with CQ-treated MG showed a reduced production of IL-6 and IL-1β and an increase in IL-10 production. Ultimately, paralysis and weakness are a result of the loss of neuronal transmission, and OL play an important role in maintaining neuronal growth and providing the myelin sheaths [59,60,61]. We observed that CQ-treated mice had more MBP^+^ OLs than PBS-treated mice (not shown). We did not investigate whether CQ induces myelination directly or if this finding is a by-stander result of reduced inflammation in the CNS, which warrant further investigations.

Overall, results presented in this study clearly show that CQ suppresses ongoing neuroinflammation by acting in both leukocytes and CNS resident cells. This finding places CQ as a promising therapeutic agent due to its modulation of both inflammation and neurodegeneration, and strengthen the basis for the use of CQ in patients with MS. The majority of FDA-approved drugs to treat MS target leukocytes and inflammation with limited effect on CNS resident cells [62,63,64]. Moreover, CQ has high bioavailability when taken orally [65,66], which increases acceptability among patients. Thus, we believe that CQ would be as efficient as, if not better than, current FDA-approved drugs in the treatment of MS.

## 5. Conclusions

Our results show that CQ is a potent modulator of the immune system and CNS resident cells. Moreover, CQ suppressed EAE progression and interfered with the activation of leukocytes and MG. CQ-treated MG-derived cytokines modulated T cells and astrocytes towards an anti-inflammatory phenotype that likely influenced the inflammation in the CNS. Overall, our data reveal a novel pathway by which CQ suppresses neuroinflammation that can be harnessed to develop novel strategies to treat MS.
